# Benzodiazepine prescription and length of hospital stay at a Japanese university hospital

**DOI:** 10.1186/1751-0759-3-10

**Published:** 2009-10-09

**Authors:** Mutsuhiro Nakao, Mikiya Sato, Kyoko Nomura, Eiji Yano

**Affiliations:** 1 Department of Hygiene and Public Health, Teikyo University School of Medicine, Tokyo, Japan; 2 Division of Psychosomatic Medicine, Teikyo University Hospital, Tokyo, Japan

## Abstract

**Background:**

The relationship between bed days and benzodiazepine prescription (BDZ) in Western countries is inconclusive, and no hospital-based report has documented this phenomenon in Japan. This study was done to assess the association between bed days and BDZ in a Japanese hospital.

**Methods:**

21,489 adult patients (55.1% men, mean age 59.9 years old) hospitalized between April, 2005 and December, 2006 were enrolled in the study. Patient age, sex, ICD-10 diagnosis, prescription profile, and days of hospital stay were assessed in 13 non-psychiatric departments using a computer ordering system. Patients prescribed a benzodiazepine during hospitalization were defined as positive.

**Results:**

Of the total sample, 19.9% were allocated to the benzodiazepine (+) group. Female sex and older age were significant factors associated with benzodiazepine prescription. The median number of bed days was 13, and the likelihood of BDZ significantly increased with the number of bed days, even after controlling for the effects of age, gender, and ICD-10 diagnosis. For example, when the analysis was limited to patients with 50 bed days or longer, the percentage of BDZ (32.7%) was equivalent to that of a report from France.

**Conclusion:**

Irrespective of department or disease, patients prescribed benzodiazepine during their hospital stay tended to have a higher number of bed days in the hospital. The difference in the prevalence of BDZ between this study and previous Western studies might be attributed to the relatively short length of hospital stay in this study. Because BDZs are often reported to be prescribed to hospitalized patients without appropriate documentation for the indications for use, it is important to monitor the rational for prescriptions of benzodiazepine carefully, for both clinical and economical reasons.

## Background

Benzodiazepines are among the most commonly used classes of medication because of their multiple therapeutic actions as anxiolytics, sedative hypnotics, anticonvulsants, and muscle relaxants [1,2]. However, it has been reported that benzodiazepines are often prescribed in hospitals and for surgical patients in the absence of appropriate documentation of the indications for their use [3-7]. Benzodiazepine use during hospital stays is an important factor in the excessive long-term use of these drugs and can be harmful especially to the elderly, who have increased risks of falls and fractures [8,9] and cognitive and memory changes [10,11] that could dramatically decrease their quality of life. Thus it is important to prescribe benzodiazepines for inpatients appropriately, considering the balance of benefit and risk.

Although the relationship between bed days and benzodiazepine prescription has been studied in several Western countries, the number of subjects was relatively small (<1,000) [12-16], the analysis was limited to the elderly [12,13,17] or to those at limited departments [12,14,15], or the diagnostic information was not controlled for in the analyses [4,18]. The findings are inconsistent among such Western studies, and no empirical study has been conducted on these issues in Japanese hospitals.

Thus we quantitatively clarified the relationship between benzodiazepine prescription and length of hospital stay at our Japanese university hospital, taking into consideration the effects of important clinical factors; patient age, sex, and diagnosis; in this study.

## Methods

### Study setting

The study population consisted of 21,489 adult inpatients aged 18 years or older, who were admitted to 13 departments at Teikyo University Hospital, Tokyo, Japan: internal medicine, neurology, general surgery, neurosurgery, cardiac surgery, orthopedics, plastic surgery, obstetrics and gynecology, ophthalmology, otorhinolaryngology, dermatology, urology, and emergency medicine. The hospital is a tertiary care hospital with 1,154 hospital beds, and approximately 60,000 outpatients visit each year [19-22]. Of the 15 departments in the hospital, the Department of Psychiatry was excluded because of the study purpose, and Pediatrics was excluded because the study was targeted at adults. All clinical activities, including prescriptions and length and monetary cost of hospital stay, are recorded in a computer ordering system (COS) at the hospital. To create a patient-based data set, data syntheses were conducted using Access 2000 and a SQL server 2000 (Microsoft, Inc., Japan). Diagnoses were based on ICD-10 categories, and physicians-in-charge entered the three-digit ICD-10 codes. The diagnostic categories analyzed were as follows: infectious diseases (A00-B99), neoplasms (C00-D48), blood diseases (D50-D89), endocrine diseases (E00-E90), nervous system diseases (G00-G99), eye diseases (H00-H59), ear diseases (H60-H95), circulatory diseases (I00-I99), respiratory diseases (J00-J99), digestive diseases (K00-K93), skin diseases (L00-L99), musculoskeletal diseases (M00-M99), genitourinary diseases (N00-N99), obstetrical diseases (O00-O99), other symptoms and signs (R00-R99), and other diseases (remaining categories). The study period was from 1 April 2005 to 31 December 2006.

The study was carried out in accordance with the Declaration of Helsinki (1981) of the World Medical Association. The study protocol was approved by the Ethics Committee of Teikyo University School of Medicine.

### Benzodiazepine prescription

Referring to the Anatomical Therapeutic Chemical Classification System [23], index groups included N05BA (benzodiazepine derivatives in anxiolytics, 01 = diazepam, 02 = medazepam, 04 = oxazolam, 06 = lorazepam, 08 = bromazepam, 12 = alprazolam, 18 = ethyl loflazepate, 19 = etizolam, 21 = clothiazepam, 22 = cloxazolam, 23 = tofisopam), N05CD (benzodiazepine derivatives in hypnotics and sedatives, 01 = flurazepam, 02 = nitrazepam, 03 = flunitrazepam, 05 = triazolam, 06 = lormetazepam, 09 = brotiazepam, 10 = quazepam), and N5CM (other hypnotics and sedatives, rilmazafone hydrochloride). Information was obtained regarding benzodiazepine prescription during the hospital stay. Of the 8,017,817 tablets and capsules prescribed in the study period, the total number of benzodiazepines was 349,449 (4.4%). The most frequently prescribed benzodiazepines, in descending order, were brotiazepam (n = 74,408), etizolam (n = 58,385), triazolam (n = 39,545), lorazepam (n = 28,056), flunitrazepam (n = 25,026), nitrazepam (n = 23,820), diazepam (n = 19,000), alprazolam (n = 17,515), ethyl loflazepate (n = 17,208), and bromazepam (n = 12,351).

The patients were divided into two groups depending on the prescription of benzodiazepines: the benzodiazepine (+) group consisted of those with any prescription for benzodiazepines during their hospital stay; the benzodiazepine (-) group had no benzodiazepine prescription during the hospital stay.

### Data analysis

Data were analyzed using the SAS statistical package [24], and *P *< 0.05, all two-tailed, was deemed to be statistically significant. Adjustment for age (continuous variable), sex (women or men), and ICD-10 diagnosis (16 categories) was done by multiple logistic regression analysis. The odds ratio for benzodiazepine prescription was estimated according to the distribution of bed days (4 days for the 25^th ^percentile, 13 days for the median, and 49 days for the 75^th ^percentile). The data on bed days were divided into four ordinal categories because their distribution was not assumed to be normal (P = 0.010 for Kolmogorov-Smirnov test).

## Results

**Figure 1:**
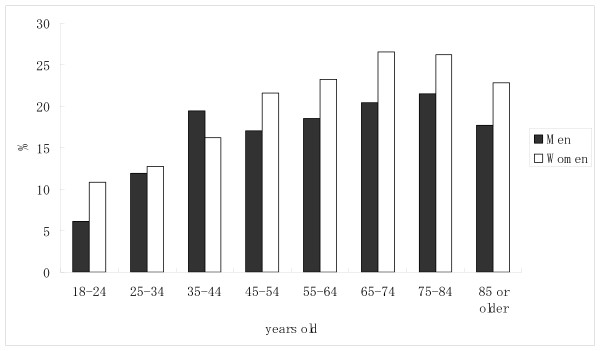
**Age- and gender-specific percentages of patients prescribed benzodiazepines**. The number of male inpatients in each age category was as follows: 18--24 yr (n = 391), 25--34 yr (n = 827), 35--44 yr (n = 1017), 45--54 yr (n = 1119), 55--64 yr (n = 2547), 65--74 yr (n = 3285), 75--84 yr (n = 2207), and ≥ 85 yr (n = 451). The number of female inpatients in each age category was as follows: 18--24 yr (n = 399), 25--34 yr (n = 1241), 35--44 yr (n = 1148), 45--54 yr (n = 843), 55--64 yr (n = 1519), 65--74 yr (n = 2070), 75--84 yr (n = 1807), and ≥ 85 yr (n = 618). * P < 0.05 between sexes (chi-square test, two-tailed, Bonferroni multiple comparisons).

**Figure 2:**
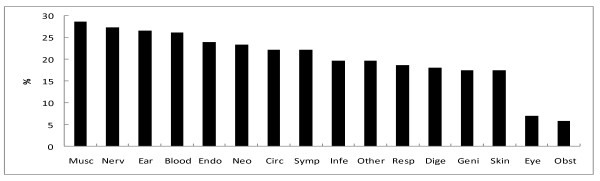
**Percentage of patients prescribed benzodiazepines according to ICD-10 category**. Abbreviations of ICD-10 categories are as follows; "Musc" for musculoskeletal diseases (n = 859), "Nerv" for nervous system diseases (n = 931), "Ear" for ear diseases (n = 526), "Blood" for blood diseases (n = 126), "Endo" for endocrine diseases (n = 699), "Neo" for neoplasms (n = 5,321), "Circ" for circulatory diseases (n = 3,221), "Symp" for other symptoms and signs (n = 298), "Infe" for infectious diseases (n = 550), "Other" for other diseases (n = 1,626), "Resp" for respiratory diseases (n = 1,188), "Dige" for digestive diseases (n = 1,883), "Geni" for genitourinary diseases (n = 1,141), "Skin" for skin diseases (n = 235), "Eye" for eye diseases (n = 2,094), and "Obst" for obstetrical diseases (n = 791).

**Figure 3:**
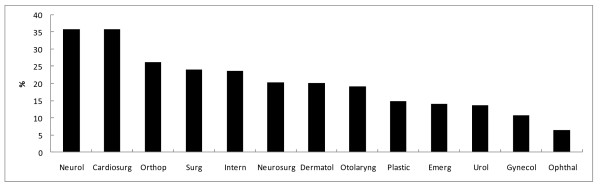
**Percentage of patients prescribed benzodiazepines by departments**. Abbreviations of departments are as follows; "Nuerol" for neurology (n = 520), "Cardiosurg" for cardiac surgery (n = 243), "Orthop" for orthopedics (n = 1,378), "Surg" for general surgery (n = 3,394), "Intern" for internal medicine (n = 6,811), "Neurosurg" for neurosurgery (n = 901), "Dermatol" for dermatology (n = 258), "Otolaryng" for otorhinolaryngology (n = 1,371), "Plastic" for plastic surgery (n = 430), "Emerg" for emergency medicine (n = 752), "Urol" for urology (n = 1,760), "Gynecol" for obstetrics and gynecology (n = 1,532), and "Ophthal" for ophthalmology (n = 2,139).

The number of inpatients prescribed benzodiazepine was 4,282 (19.9%). The percentage of patients prescribed benzodiazepine was significantly higher for women than for men, and the percentage increased with age (Table 1). When divided into 10-year age categories, the percentage of patients prescribed benzodiazepine tended to increase with age, both for men and women (Figure 1). The percentage of patients prescribed benzodiazepine by ICD-10 diagnosis is shown in Figure 2. When the specific three-digit codes of ICD-10 were analyzed (n > 100), benzodiazepine prescription was most common for patients diagnosed with chronic renal failure (N18, 31.3%), followed by heart failure (I50, 29.3%), acute myocardial infarction (I21, 28.9%), malignant neoplasm of the liver and intrahepatic bile ducts (C22, 28.0%), post-procedural disorders of the nervous system (G97, 28.0%), malignant neoplasm of the bronchus and lung (C34, 27.7%), non-insulin-dependent diabetes mellitus (E11, 25.0%), and malignant neoplasm of the bladder (C67, 22.2%). The percentage of patients prescribed benzodiazepine by department is shown in Figure 3. It was highest at the department of neurology (35.8%), followed by cardiac surgery (35.8%), orthopaedics (26.3%), general surgery (24.1%), internal medicine (23.7%), neurosurgery (20.3%), and dermatology (20.2%).

**Table 1:** Odds ratios (95% confidence intervals, C.I.) for benzodiazepine prescription (BDZ) to 21,489 inpatients

		**N**	**%BDZ**	**Odds ratio**	**(95%C.I.)**^**a**^
Age, years					
	18 to 44	5,023	14.0	---	
	45 to 64	6,028	19.8	1.51	(1.37, 1.68)
	65 to 74	5,355	22.7	1.80	(1.63, 2.00)
	75 or older	5,083	23.0	1.83	(1.65, 2.03)

Sex					
	Male	11,844	18.6	---	
	Female	9,645	21.6	1.21	(1.13, 1.29)

Number of bed days					
	4 or shorter	5,938	9.1	---	
	5 to 13	5,258	16.5	1.98	(1.76, 2.22)
	14 to 49	4,917	22.8	2.96	(2.65, 3.31)
	50 or longer	5,376	32.7	4.86	(4.37, 5.40)

^a^The variables age and sex from logistic regression analysis were used to estimate a crude odds ratio for benzodiazepine prescription.

For the number of bed days, multiple logistic regression analysis was used to estimate the odds ratio for benzodiazepine use, adjusted by age, sex, and ICD-10 diagnosis.

The arithmetic mean (standard deviation) for bed days was 29.6 (35.7) days, the geometric mean 13.9 (4.0) days, and the median value (range) was 13 (1-254) days for the total sample. The percentage of patients prescribed benzodiazepine tended to increase with the number of bed days. According to the results of multiple logistic regression analysis, benzodiazepine prescription was significantly associated with bed days, even after adjusting for the effects of the three independent variables; age, gender, and ICD-10 diagnosis (Table 1).

## Discussion

One-fifth of the inpatients had been prescribed benzodiazepines during their hospital stay. The elderly (≥ 65 years) were more likely to have a benzodiazepine prescribed compared to the non-elderly, as were women compared to men. Although the percentage of benzodiazepine prescribed varied by department and ICD-10 diagnosis, it was significantly associated with a higher number of bed days, even after adjusting for the effects of age, gender, and ICD-10 diagnosis.

The percentage for benzodiazepine prescription was higher than was found for outpatients in our previous study (12%) [19,20], but was rather low when compared with Western studies of inpatients, which have reported prevalences of 33-45% [3,15,18]. A possible explanation for this difference is the relatively short length hospitalization in our study. When the analysis was limited to those with ≥ 50 bed days, the percentage given benzodiazepine prescriptions was equivalent to that in a report from France (33%) [15]. However, this fact does not necessarily weaken the generalizability of our results to Japanese inpatients, because the arithmetic mean of bed days is reported to be 20 days for Japanese general hospitals and 36 days for all Japanese hospitals according to a national survey by the Japanese Ministry of Health, Labor, and Welfare [25]. The average number of bed days in our hospital (= 30 days) was typical of Japanese hospitals.

Another possible explanation is the Japanese unwillingness to admit to psychiatric symptoms. The Japanese people have high thresholds for not visiting psychiatric clinics [26] and tend to complain of somatic symptoms, even if they suffer from mental illnesses such as depression [27,28]. There may have been some patients in our study for whom psychological complaints were masked and who were followed up without anti-psychotropic regimens. It is reasonable that specific diseases such as musculoskeletal and nervous system diseases and the corresponding departments of neurology and orthopedics had high prevalences of benzodiazepine prescription because increased muscle tension is often accompanied by psychological distress, and benzodiazepines have a therapeutic effect as a muscle relaxant [1,2,29]. It is also reasonable that patients hospitalized in the department of cardiac surgery had a higher prevalence of benzodiazepine prescription because of cardiovascular symptoms due to their disease and the extremely anxious situation before heart surgery [30]. Although these implications are inconclusive, extensive intervention would be needed for inpatients with such disease conditions and further study needs to be done to assess their psychological distress and the degree of patient satisfaction. Through such assessment, important factors may be found for how to shorten the number of bed days for such patients.

Because an increased number of bed days has been reported to be closely related to the anxiety of inpatients with a wide rage of general medical disorders [10], it is possible that those staying at a hospital for a long time are likely to be anxious about their disease condition and that such worries may lead the patient seeking to use benzodiazepines. This was a cross-sectional study, and it is also possible that the side effects of benzodiazepines may contribute to extended hospital stays [31]. There may be room for reducing medical costs by targeting patients who have been hospitalized for a long time [32,33]. Although the data are preliminary and not listed in the table, the percentage prescribed benzodiazepine was 10.2% for patients with hospital expenses of less than 2,000 US$ (n = 5,562), whereas it was 32.0% for those with expenses of 8,500 US$ or more (n = 5,169). For example, it may be good to routinely screen the mental condition of inpatients using a simple questionnaire before prescribing benzodiazepines and to use a consultation-liaison service with psychiatric specialists for the treatment of patients prescribed benzodiazepines [34]. Particularly, the elderly should be targeted because of the high prevalence of benzodiazepine prescription to those aged = 65 years. Possible side effects of benzodiazepines, such as memory loss and increased risks for falls and fractures, are also issues of the elderly [8,9,11,13,35].

Before making conclusions, we must mention several study limitations. First, this was a database study, and the specific reasons for prescribing benzodiazepine were often unclear. For example, it is possible that some inpatients in the department of surgery may have been prescribed benzodiazepine as a pre-medication. However, a close relationship between benzodiazepine prescription and bed days was observed in non-surgical departments as well. Future tasks would be to gather information on the use of benzodiazepines from both inpatients and the physician-in-charge. Second, a cross-sectional study design prevented us from coming to conclusions regarding causal relationships between benzodiazepine prescription and the number of bed days. The use of benzodiazepine might be a simple marker for, rather than a cause of, prolonged hospitalization. That is, the chance of benzodiazepine prescription would be higher for patients with longer hospital stays because the percentage of benzodiazepine use increased with increased bed days. However, we can at least state that the monitoring of benzodiazepine prescription is important in hospitals, whether or not benzodiazepine prescriptions are a cause or a consequence. For those who have already been in the hospital for long time, the continuing appropriateness of benzodiazepine prescription should be checked. Finally, our findings cannot be compared with those at other medical institutions in Japan because, to our knowledge, this is the first study of its type in Japan. The hospital of the study is a university teaching hospital that has hundreds of physicians who work in all general departments that offer outpatient clinics; thus, any tendency in prescribing practices might reflect the effects of Japanese educational conferences and the advice of supervisors, rather than individual prescribing preferences [36]. To generalize our conclusions requires further studies at other Japanese institutions, as well as hospitals in other countries.

## Conclusion

Despite these limitations, our findings have valuable implications. This is the first study to report data on inpatients that are relevant to the discussion of the use of benzodiazepines in Japanese hospitals. We found that the percentage of benzodiazepine prescriptions increased with the number of bed days and that patients prescribed benzodiazepines generally stayed for a longer period than those who were not prescribed benzodiazepines. Based on the finding of a high percentage of benzodiazepine prescription to elderly inpatients, pharmacological treatment should be considered carefully using evidence-based clinical guidelines; alternatively, physicians might refer patients to psychiatrists or specialists in mental health. Further studies are needed to determine the precise cause(s) of the high level of benzodiazepine prescription in Japanese hospitals.

## Abbreviations

(COS): computer ordering system; (ICD-10): International Classification of Diseases, 10^th ^Edition; (SAS): Statistical Analysis System.

## Competing interests

The authors declare that they have no competing interests.

## Authors' contributions

MN and MS contributed to the data collection and analysis for the study. KN and KN contributed to the completion of the study design, and EY was consulted throughout the process of designing and writing the paper. All four authors attended discussion meetings that contributed to the completion of the paper.
